# Cryopreserved fascia lata allograft use in surgical facial reanimation: a retrospective study of seven cases

**DOI:** 10.1186/s40902-020-0246-9

**Published:** 2020-02-08

**Authors:** Francesco Silan, Fabio Consiglio, Francesco Dell’Antonia, Giulia Montagner, Diletta Trojan, Giorgio Berna

**Affiliations:** 1 Plastic and Reconstructive Surgery Department, Ca’ Foncello Hospital, 31100 Treviso, Italy; 2 Fondazione Banca dei Tessuti di Treviso Onlus, Via dell’Ospedale 3, 31100 Treviso, Italy

**Keywords:** Fascia lata, Cryopreservation, Facial palsy, Homograft, Orthodromic technique

## Abstract

**Background:**

Facial palsy treatment comprises static and dynamic techniques. Among dynamic techniques, local temporalis transposition represents a reliable solution to achieve facial reanimation. The present study describes a modification of the temporalis tendon transfer using a cryopreserved fascia allograft.

**Case presentation:**

Between March 2015 and September 2018, seven patients with facial palsy underwent facial reanimation with temporalis tendon transfer and fascia lata allograft. Patients with long-term palsy were considered, and both physical and social functions were evaluated. The mean follow-up time was 21.5 months. No immediate complications were observed. Patients reported improvement in facial symmetry both in static and dynamic. Improvement was noticed also in articulation, eating, drinking, and saliva control. The Facial Disability Index revealed an improvement both in physical function subscale and in the social/well-being function subscale.

**Conclusions:**

This modified orthodromic technique allows to reduce the operative time and the risk of complications connected to the use of autologous tissues. The use of the cryopreserved fascia allografts from cadaveric donors seems to provide promising and long-standing results in the treatment of facial palsy.

## Background

Facial palsy is an invalidating condition. In fact, besides the reduction of physical function, it affects social interaction. Facial reanimation aims to ameliorate facial symmetry and the ability to smile, but the recovery of a totally normal facial function is difficult to achieve. Many techniques have been described to treat long-standing facial paralysis: these can be static or dynamic. Among dynamic techniques, free gracilis flap represents, for many authors, the favorite technique due to the possibility of achieving spontaneous smiles in patients with hemifacial palsy [1, 2]. When it is not possible to perform a microsurgery flap or when the patient refuses a microsurgical procedure, local muscle transposition can provide a reliable solution. Temporalis muscle flap is often used to restore the symmetry and to reanimate the midface. Both gracilis muscle free flap transfer and temporalis tendon transfer techniques improve mouth symmetry at rest and during smile, but a better excursion was observed with gracilis free muscle transfer [3]. Many variants of temporalis transposition have been described, using it in an orthodromic or antidromic manner, with or without the use of a fascia sling [4–8]. The present study describes a modification of the orthodromic technique using a cryopreserved fascia lata allograft. The procedure avoids donor site-related morbidity and complications. The cases of seven patients with unilateral facial palsy operated between 2015 and 2018 are reported.

## Case presentation

Between March 2015 and September 2018, seven patients with long-term facial palsy underwent facial reanimation with temporalis tendon transfer and fascia lata allograft at the Plastic and Reconstructive Surgery Department of Treviso Hospital. The diagnosis was based on clinical history and physical examination. An eye exam was performed in those patients who had lagophthalmos. Physical examination revealed facial asymmetry, drooping of the paralyzed side, and flattening of the nasolabial sulcus. Patients showed an inability to pucker their lips or smile. All patients had long-term facial palsy: the mean time since the onset of the paralysis was 5.43 years (range 2–20 years). However, electromyography was performed in all patients to assess atrophy of facial muscles. Five patients were female and two patients male. The mean age at the time of the surgery was 54.86. The etiology of facial palsy was heterogeneous. In Table 1, patients’ de-identified demographic and clinical data are reported.

**Table 1:** Demographical and clinical data

Case	Age	Sex	Cause of palsy	Duration of palsy (years)
1	69	F	Parotidectomy	2
2	45	M	Trauma	2
3	79	F	Neurovascular conflict	20
4	58	F	Herpes simplex virus	5
5	45	F	Acoustic neuroma	3
6	45	F	Acoustic neuroma	3
7	43	M	Unknown	3

**Fig. 1:**
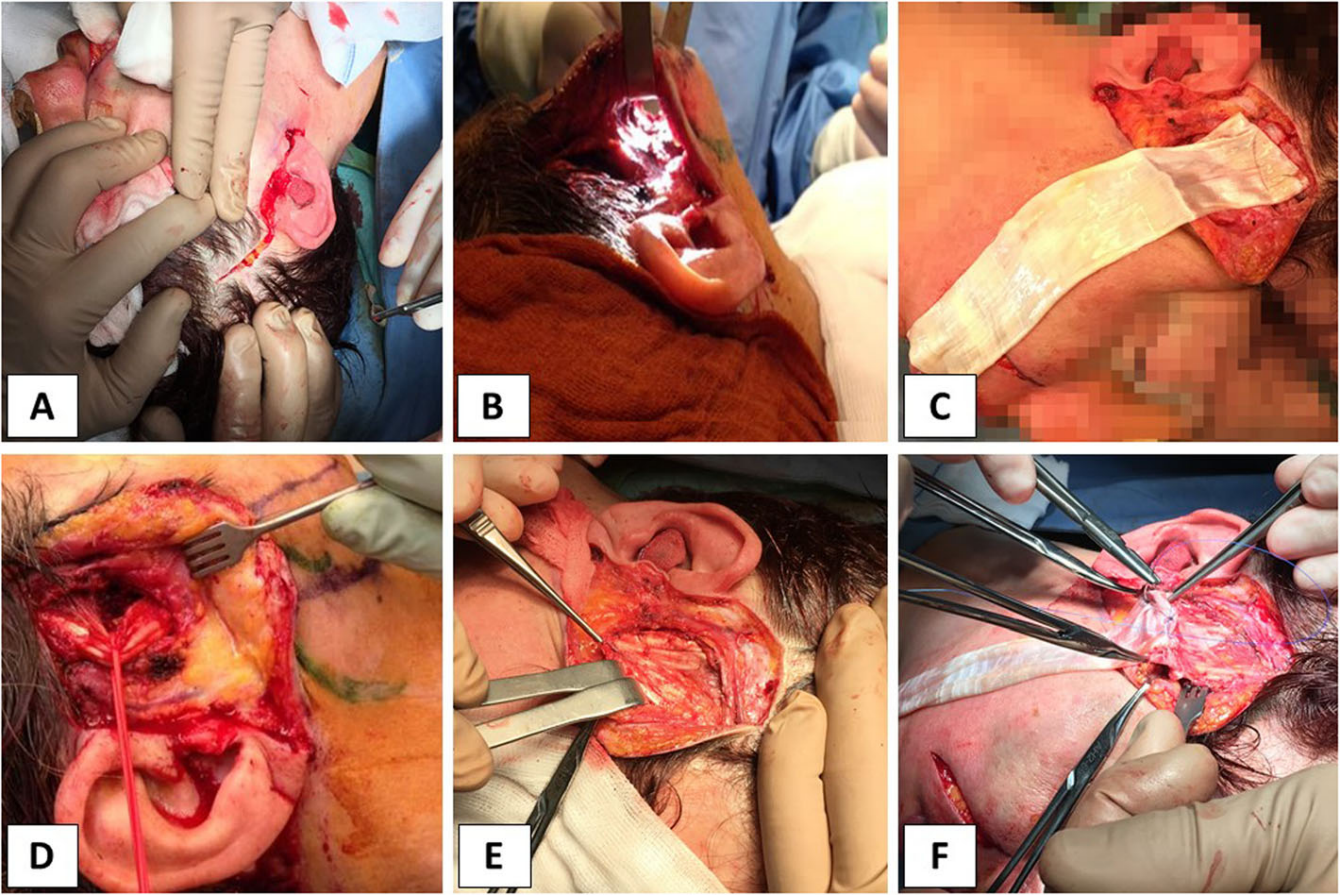
Intraoperative images. **a** Preauricular incision extending to postauricular and temporal area. **b** Subcutaneous tunnel. **c** Cadaveric fascia lata graft. **d** Temporalis tendon dissection. **e** Exposed temporalis tendon. **f** Cadaveric fascia lata graft sutured to the temporalis tendon

Data were collected from medical records, and the Italian version of the Facial Disability Index (FDI) questionnaire was used to evaluate facial palsy-related disabilities. FDI is a 10-item questionnaire with two subscale scores: 5 items contribute to the physical function (PF) subscale and 5 items to the social/well-being (SF) function subscale. Both subscales are transformed into a score on a 100-point scale, with 100 indicating unimpaired physical or social/well-being function [9]. The questionnaire was filled by the patients before and after surgery; all questions refer to the previous months. Before the surgical procedure, markings were drawn in standing position. The patient was asked to smile, and a smiling vector and nasolabial fold position was observed in the not paralyzed side and reported to the affected side. A pretragal incision was then marked, extending it to the postauricular and to the temporal area of the scalp (Fig. 1a). Further markings were drawn at the base of the nostril. At first, an incision along the column of the philtrum was performed, but later we noticed that it was unnecessary. Nasotracheal intubation was mandatory to avoid distortion of the lips. Both the paralyzed side and the not affected side were prepped. Before starting, infiltration with a saline solution with adrenaline was performed. Incisions were made along markings. Blunt dissection was performed in a subcutaneous plane until the nasolabial fold was reached and then incised (Fig. 1b). Further incision was performed at the base of the nostril. Fascia lata allografts were collected, processed, and cryopreserved by “Fondazione Banca dei Tessuti di Treviso,” a tissue bank, in accordance with the requirements approved by the National Transplant Centre. Donor selection includes serological tests for detecting hepatitis B and C viruses, human immunodeficiency virus, cytomegalovirus, human T cell lymphotropic virus, and the syphilis pathogen, along with polymerase chain reaction for detecting human immunodeficiency virus and hepatitis B and C viruses. All tissues were decontaminated two times with a validated antibiotic cocktail of gentamicin (Fisiopharma, Palomonte, Salerno, Italy), vancomycin (Pharmatex, Milan, Italy), and meropenem (Fresenius Kabi AG, Bad Homburg, Germany) [10, 11], and microbiological tests were performed following internal procedures. During the processing of fascia lata, adipose tissue and muscle tissue were removed. Before cryopreservation, fascia lata was transferred in low temperature–resistant ethylene-vinyl acetate bags with a solution composed of BASE medium (Alchimia Srl, Italy), 10% dimethylsulfoxide (Wak-Chemie Medical GmbH, Germany), and 10% human serum albumin (Alburex 20%, CSL Behring GmbH, Germany). Cryopreservation was achieved using a programmable cryogenic freezer (Planer KryoSave Integra, 750-30), which triggers a controlled cooling rate. Tissues were stored at − 140 °C in liquid nitrogen vapor phase and thawed before use. The thawed graft (Fig. 1c) was separated in two slings, one larger (3–4 cm) for the nasolabial fold and the other narrower (1 cm) for the nostril. Cadaveric fascia lata graft was sutured to the dermis of nasolabial fold and nostril with 4/0 nylon suture. Traction on the slings was performed to reach the right grade of correction. The grafts were then sutured to the exposed temporalis tendon with 4/0 nylon suture and redundant grafts were excised (Fig. 1d–f). Any redundant skin was excised before closing it. No immediate complications were observed and the improvement of facial symmetry was noticed immediately. Patients started specific physical therapy 1 month after surgery.

### Case 1

A 69-year-old woman developed a facial palsy on the left side 2 years before, after parotidectomy. She had sagging of the eyebrow and lagophthalmos. Treatment with lubricating eye drops and eye taping was undertaken until surgery. No ancillary procedure was performed before surgery. After surgery, an edema developed on the operated side. However, both PF and SF increased after surgery as reported in Table 2. Follow-up lasted 14 months and no complications were observed.

**Table 2:** Physical function (PF) and social/well-being function (SF) patient scores pre- and post-operation

Case	PF pre	PF post	SF pre	SF post
1	70	75	68	88
2	45	65	48	60
3	45	50	56	48
4	30	70	52	88
5	35	70	52	88
6	35	85	36	88
7	45	80	60	72

### Case 2

**Fig. 2:**
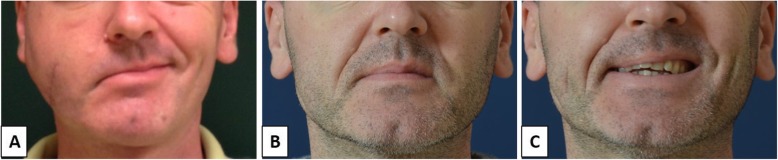
Facial palsy secondary to trauma (patient 2). **a** Preoperative aspect, smiling. **b** Six months postoperative. **c** Six months postoperative, smiling

Patient 2 was a 45-year-old man with a facial palsy on the right side, secondary to trauma. He had sagging of the eyebrow and lagophthalmos. Treatment with lubricating eye drops and eye taping was undertaken until surgery. Gold weight insertion with lateral canthoplasty and brow lift were performed as an ancillary procedure. No complications were observed immediately after surgery and during the 1-year follow-up. FDI questionnaire revealed an improvement of both SF and PF scores (Table 2). Figure 2 illustrates the aspect of patient 2 before surgery and 6 months after surgery.

### Case 3

A 79-year-old woman developed a facial palsy on the left side 20 years before due to a neurovascular conflict. She had sagging eyebrow and lagophthalmos. Lubricating eye drops and eye taping were prescribed until surgery. Gold weight insertion with lateral canthoplasty was performed during surgery. After surgery, facial symmetry ameliorated but only PF increased. However, follow-up lasted 9 months and no complications were observed.

### Case 4

A 58-year-old woman had a facial palsy on the right side 5 years before. She had no relevant surgeries in the past. Gold weight insertion was performed as an ancillary procedure. No immediate complications were observed immediately after surgery and up to 22 months after surgery. Improvement of both PF and SF was recorded (see Table 2).

### Cases 5 and 6

**Fig. 3:**
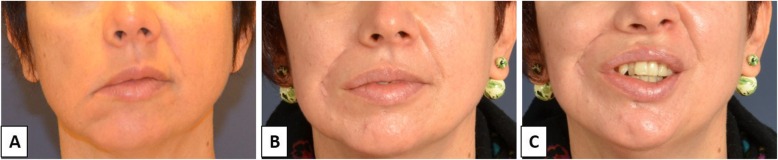
Facial palsy secondary to acoustic neuroma (patient 6). **a** Preoperative aspect. **b** Six months postoperative. **c** Six months postoperative, smiling

Patients 5 and 6 were both 45-year-old women with facial palsy on the right side. The palsy developed 3 years before because of acoustic neuroma that was operated in the past. Patient 5 had also sagging eyebrow and lagophthalmos. In these cases, gold weight insertion was performed as an ancillary procedure. Improvement of both PF and SF was recorded after surgery, as reported in Table 2. No complications were observed during the follow-up that lasted 42 and 30 months for patients 5 and 6, respectively. Clinical aspects of patient 6 before surgery (a) and 6 months after surgery (b and c) are reported in Fig. 3.

### Case 7

A 43-year-old man had a facial palsy on the left side with unknown etiology. The palsy appeared 2 years before but no relevant comorbidities were reported. No ancillary procedures were performed. After surgery, fascia lata detachment occurred; however, postoperative PF and SF scores increased because of the improvement in facial symmetry. No other complications were observed 22 months after surgery.

## Discussion

We report seven cases of facial palsy in which increased facial symmetry was achieved, performing a partial temporalis tendon transfer with the use of cryopreserved fascia lata allograft. The increased facial symmetry at rest was noticed immediately after surgery, especially after edema resolution. Facial reanimation was achieved later, thanks to proper and specific physical therapy and at-home exercises. Best results were noticed in patients who adhered strictly to the physical therapy program. Only one patient had an unsatisfactory result due to inappropriate postoperative physical therapy. Patients reported improvement of facial symmetry, both in static and dynamic. Improvement was noticed also in articulation, eating, drinking, and saliva control. FDI questionnaire revealed the amelioration of both PF and SF after surgery. Only one patient reported an improvement in the PF subscale but a worsening in the SF subscale. In one case, fascia lata detachment occurred; however, PF and SF increased. In fact fascia lata sling is a well-known procedure to achieve static rehabilitation in the paralyzed face. The use of fascia lata in facial palsy reanimation was first reported by Sir Gillies in 1934. He described the creation of a temporal muscle turnover flap connected to the mouth on the paralyzed side by an autologous fascia lata graft [12]. In 1953, McLaughlin introduced a modification of Gillies’s technique, detaching the insertion of the temporal muscle by an osteotomy of the coronoid process, lengthening the muscle with a fascia lata graft attached to the lower and upper lip [13]. In 1996, Breidahl et al. reported a modification of the temporalis muscle transfer using an autologous fascia lata sling. The authors stripped the tendon of the temporalis after doing an osteotomy of the zygomatic arch but avoiding a sectioning of the coronoid process. Fascia lata was then sutured to the released tendon and secured to the non-paralyzed side of the upper and lower lip [6]. More recently, Pidgeon et al. described a partial temporalis tendon transfer with an autologous fascia lata sling, where only a specific segment of the tendon is used. An osteotomy of the zygomatic arch is required to access the tendon, but sectioning of the coronoid is avoided [7]. The technique reported in this retrospective case series allows a partial temporalis tendon transfer avoiding any osteotomy. Temporalis muscle belly is bluntly dissected underneath the zygomatic arch towards the coronoid process. When the tendon is visualized, it is partially sectioned and raised superficially. Direct exposure of the coronoid process is not needed. The absence of any osteotomy in this technique allows a reduction of the operative time and a lower risk of complications. The use of banked fascia lata graft has been described for many purposes: reconstruction of osteo-dural defect in neurosurgery, ptosis surgery, urinary incontinence, and rotator cuff tears repair [14–18]. Harvesting of autologous fascia lata can determine postoperative hematoma, wound infection, muscle herniation, weakness of hip flexion, numbness, pain, superficial phlebitis, and cosmetic concern due to the scar [19]. Several options are available for rehabilitation of chronic facial nerve paralysis. The described technique achieves restoring facial dynamic reanimation in a single procedure with fast postoperative recovery. Compared with free flaps surgery, it requires neither microsurgical skills nor a double surgical team. Transfer of temporal muscle avoids the need of any reinnervation of the paralyzed muscles, preserving contralateral facial nerve. Further, it does not require any nerve graft, avoiding donor site complications, and failure of reinnervation. With the use of cadaveric fascia lata graft, no additional scars are required and donor site-related complications are avoided. No complications related to the use of the fascia lata allograft were observed. Moreover, with this technique, satisfactory long-term results were obtained and any loss of strength related to the use of cadaveric fascia was noticed. The patient’s grade of satisfaction was high. These encouraging results can provide a starting point for determining whether the application of cryopreserved fascia lata allografts can provide a promising therapeutic alternative for the treatment of facial palsy.

## Conclusions

This study presents a partial temporalis tendon transfer and the use of cryopreserved fascia lata allograft to treat facial paralysis. The modified orthodromic technique allows reducing the operative time and the risk of complications connected to the use of autologous tissues by removing the need for a second surgical site. This case report suggests how the use of a cryopreserved fascial lata homograft from a cadaveric donor can be a valuable alternative in the treatment of facial palsy providing promising and long-standing esthetical and functional results. We suggest the need for a more scrupulous clinical trial to confirm the results of our investigation.

## Data Availability

The datasets generated and/or analyzed during the current study are available from the corresponding author on reasonable request.

## Abbreviations

FDI: Facial Disability Index
PF: Physical function
SF: Social/well-being function
